# Deep-learning-based sampling position selection on color Doppler sonography images during renal artery ultrasound scanning

**DOI:** 10.1038/s41598-024-60355-5

**Published:** 2024-05-23

**Authors:** Xin Wang, Yu-Qing Yang, Sheng Cai, Jian-Chu Li, Hong-Yan Wang

**Affiliations:** 1 Department of Ultrasound, State Key Laboratory of Complex Severe and Rare Diseases, Peking Union Medical College Hospital, Chinese Academy of Medical Science and Peking Union Medical College, No. 1, Shuaifuyuan, Dongcheng District, Beijing, 100730 China; 2 State Key Laboratory of Networking and Switching Technology, Beijing University of Posts and Telecommunications, Beijing, China

**Keywords:** Deep learning, Object detection, Renal artery ultrasound, Sampling position selection, Color Doppler sonography, Renal artery stenosis, Medical research

## Abstract

Accurate selection of sampling positions is critical in renal artery ultrasound examinations, and the potential of utilizing deep learning (DL) for assisting in this selection has not been previously evaluated. This study aimed to evaluate the effectiveness of DL object detection technology applied to color Doppler sonography (CDS) images in assisting sampling position selection. A total of 2004 patients who underwent renal artery ultrasound examinations were included in the study. CDS images from these patients were categorized into four groups based on the scanning position: abdominal aorta (AO), normal renal artery (NRA), renal artery stenosis (RAS), and intrarenal interlobular artery (IRA). Seven object detection models, including three two-stage models (Faster R-CNN, Cascade R-CNN, and Double Head R-CNN) and four one-stage models (RetinaNet, YOLOv3, FoveaBox, and Deformable DETR), were trained to predict the sampling position, and their predictive accuracies were compared. The Double Head R-CNN model exhibited significantly higher average accuracies on both parameter optimization and validation datasets (89.3 ± 0.6% and 88.5 ± 0.3%, respectively) compared to other methods. On clinical validation data, the predictive accuracies of the Double Head R-CNN model for all four types of images were significantly higher than those of the other methods. The DL object detection model shows promise in assisting inexperienced physicians in improving the accuracy of sampling position selection during renal artery ultrasound examinations.

## Introduction

Renal artery stenosis (RAS) is a prevalent underlying condition in 20–35% of patients with secondary hypertension, often leading to renovascular hypertension and renal insufficiency^1,2^. Early identification and appropriate treatment of RAS are essential, as the condition can progress rapidly to end-stage renal disease, which often necessitates surgery or revascularization therapy, leading to a significant impact on patients’ quality of life and imposing a substantial economic burden^2,3^.

Color Doppler sonography (CDS) is the preferred screening method for RAS due to its cost-effectiveness, wide availability, noninvasiveness, and lack of radiation exposure^1,4^. However, its technical complexity poses challenges, and inexperienced operators may encounter high rates of misdiagnosis^5,6^. Renal artery ultrasound examination requires the operator to select a sampling position on the CDS image to acquire spectral waveforms. The anatomical variations and complex hemodynamic changes further complicate the operation and diagnosis of renal artery ultrasound, requiring an extensive learning curve. Particularly for beginners, choosing the appropriate sampling position is difficult, contributing to operator dependence and potential misdiagnosis^5,7–9^. Studies have reported varying sensitivities (54–98%) and specificities (54–99%) for CDS in detecting RAS^4,5,10,11^. Moreover, the diagnostic accuracy of RAS is generally higher among experienced physicians with longer seniority (mean of 14.3 years) compared to those with less experience (mean of 10.0 years)^8^. In cases involving abnormal renal artery branches, with or without renal artery stenosis, doctors with higher seniority exhibit an accuracy rate that is 13.5% higher than their less-experienced counterparts^9^.

Currently the rapid development of artificial intelligence (AI) technology has promoted the wide applications of deep learning (DL) approaches in medicine. DL models have demonstrated strong capabilities in image feature extraction and disease risk prediction in numerous computer-aided diagnosis (CAD) studies^12–16^. DL approaches are widely applied in areas such as the ultrasonic diagnosis of thyroid, breast, and liver diseases^17^.

**Figure 1:**
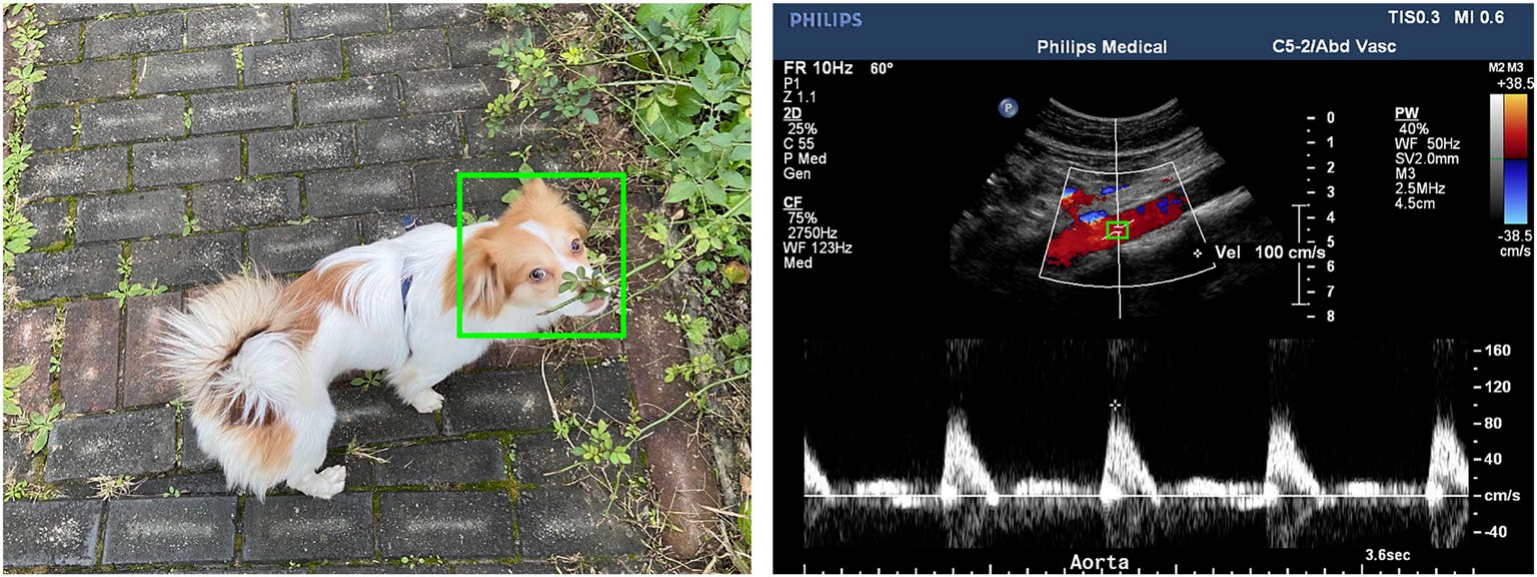
Comparison between traditional object detection and sampling position selection. The green boxes corresponded to the regions of interest, the animal face and sampling position.

In renal artery ultrasound examination, the first step is to identify the standard planes, and then select the appropriate sampling position in the CDS images (Fig. 1), which was influenced by surrounding anatomical structures and blood flow information, in order to obtain proper spectral waveforms. Appropriate sampling position selection is an indispensable step of the renal artery ultrasound examination. Currently there are few studies on AI-assisted scanning related to renal artery ultrasound examination and research on assisting sampling position selection based on DL technology has not been reported.

The annotation of sampling position on CDS images can be simplified as drawing a specific rectangular box on the image according to clinical experience (Fig. 1). By modeling above-mentioned selection process as an object detection problem in computer vision area, similar to detecting positions of animal faces in an image, our study aims to explore the validity of object detection method in the sampling position selection (Fig. 1). Possible sampling positions will be predicted automatically to assist ultrasound examination by training the deep object detection model to capture the image features, which is also a fundamental and necessary part of AI-guided renal artery ultrasound scanning.

The object detection problem involves determining whether there are instances of interested objects (e.g., nodule, lesion, and cell nucleus) within given images and returning the spatial location and confidence of each instance^18^. DL-based object detection methods can be classified into two-stage models or single-stage models. Typical two-stage models include Faster R-CNN, Cascade RCNN, and Double-Head R-CNN, etc.^19–21^. These models first extract candidate object regions and then predict the positions and categories of objects based on the candidates. Representative single-stage models include YOLO series, RetinaNet, and, the keypoint based model, FoveaBox, which treat object detection as an end-to-end regression problem^22–24^. Besides, DETR series object detection models based on the transformer structure have been proposed and achieved comparable performance with two-stage model^25^.

**Figure 2:**
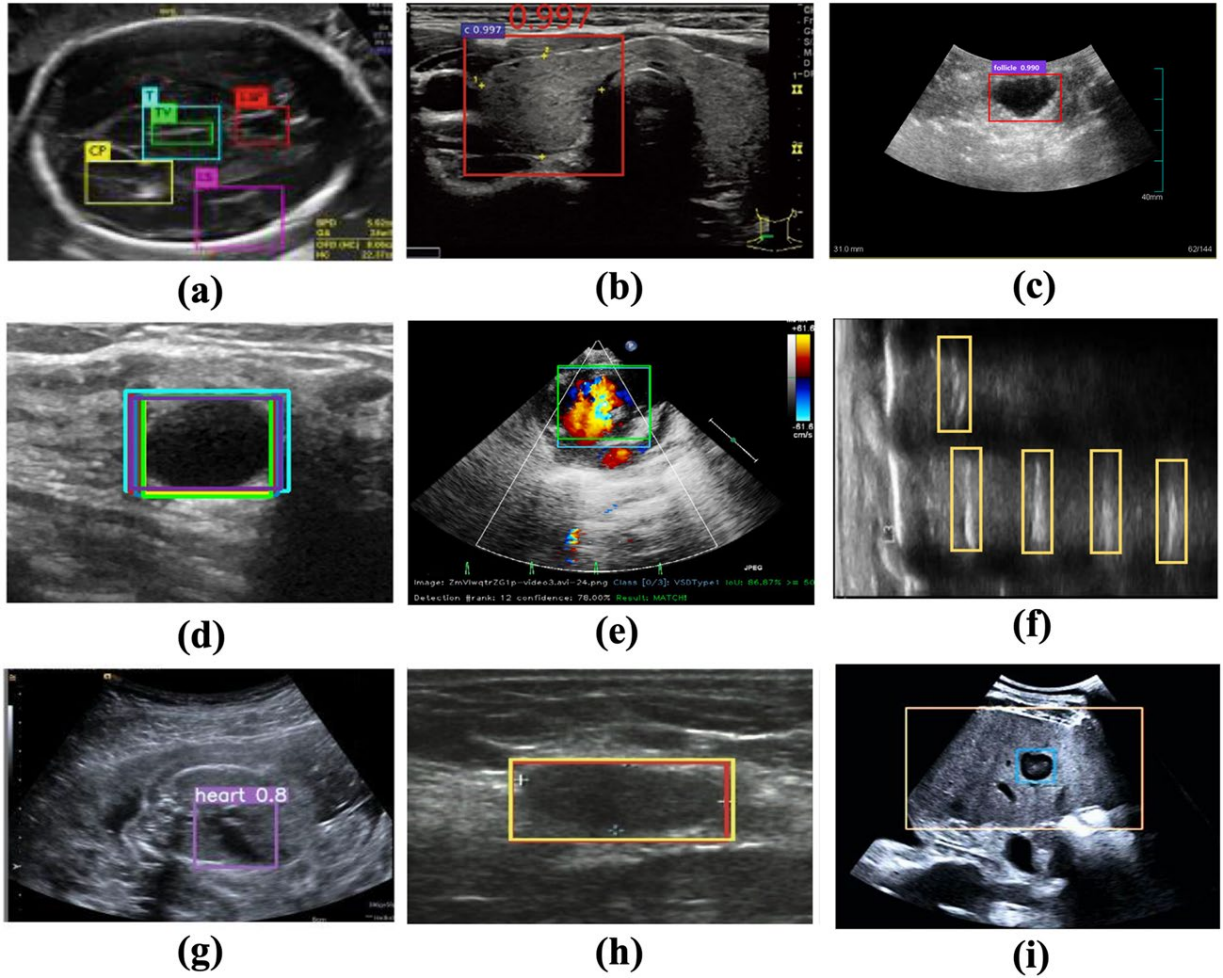
Object detection in ultrasound image analysis. (**a**) Fetal standard transthalamic plane^26^. (**b**) Thyroid papillary carcinoma^27^. (**c**) Cattle ovarian follicle^28^. (**d**) Breast benign lesion^29^. (**e**) Ventricular septal defect in echocardiographic image^30^. (**f**) Lung ultrasound feature^31^. (**g**) Anatomical plane in fetus^32^. (**h**) Breast nodule^33^. (**i**) Focal liver lesion^34^.

The application of DL object detection technology in ultrasound image analysis has been increasing, and various DL models have been proposed (Fig. 2). In 2018, Lin et al. located 5 anatomical structures within the fetal head ultrasound image using the Faster R-CNN method^26^. Li et al. modified the structure of Faster R-CNN to detect the thyroid papillary carcinoma in ultrasound images^27^. In 2019, Zeng and Liu adjusted the Faster R-CNN structure to detect cattle ovarian follicle in the follicle ultrasound image^28^. Cao et al. compared multiple DL object detection models on breast lesion detection^29^. In 2021, Chen et al. modified the single-stage detector YOLOv4 to recognize the ventricular septal defect in echocardiographic images^30^. Bassiouny et al. compared Faster R-CNN and RetinaNet in discriminate lung ultrasound feature^31^. Besides, Faster R-CNN and YOLOv5 models were also applied in detecting breast nodules and recognizing fetal anatomical plane^32,33^. Recently Dadoun et al. tried the DETR to locate the focal liver lesions in abdominal ultrasound image, and the performance of DETR preceded the Faster R-CNN^34^. It could be seen that Faster R-CNN or YOLO series models are relatively popular and different types of models are needed to be compared to select the most suitable model.

One notable difference between traditional object detection problem and the sampling position selection, is that patterns of human/animal faces are usually specific and distinct with the natural background (Fig. 2). However, area of the sampling position can be similar to its surrounding region belonging to the same artery. The selection of sampling position relies on the physician’s comprehensive analysis and clinical experience, which is a challenge for the model construction and predictive accuracy of DL object detection technology.

**Figure 3:**
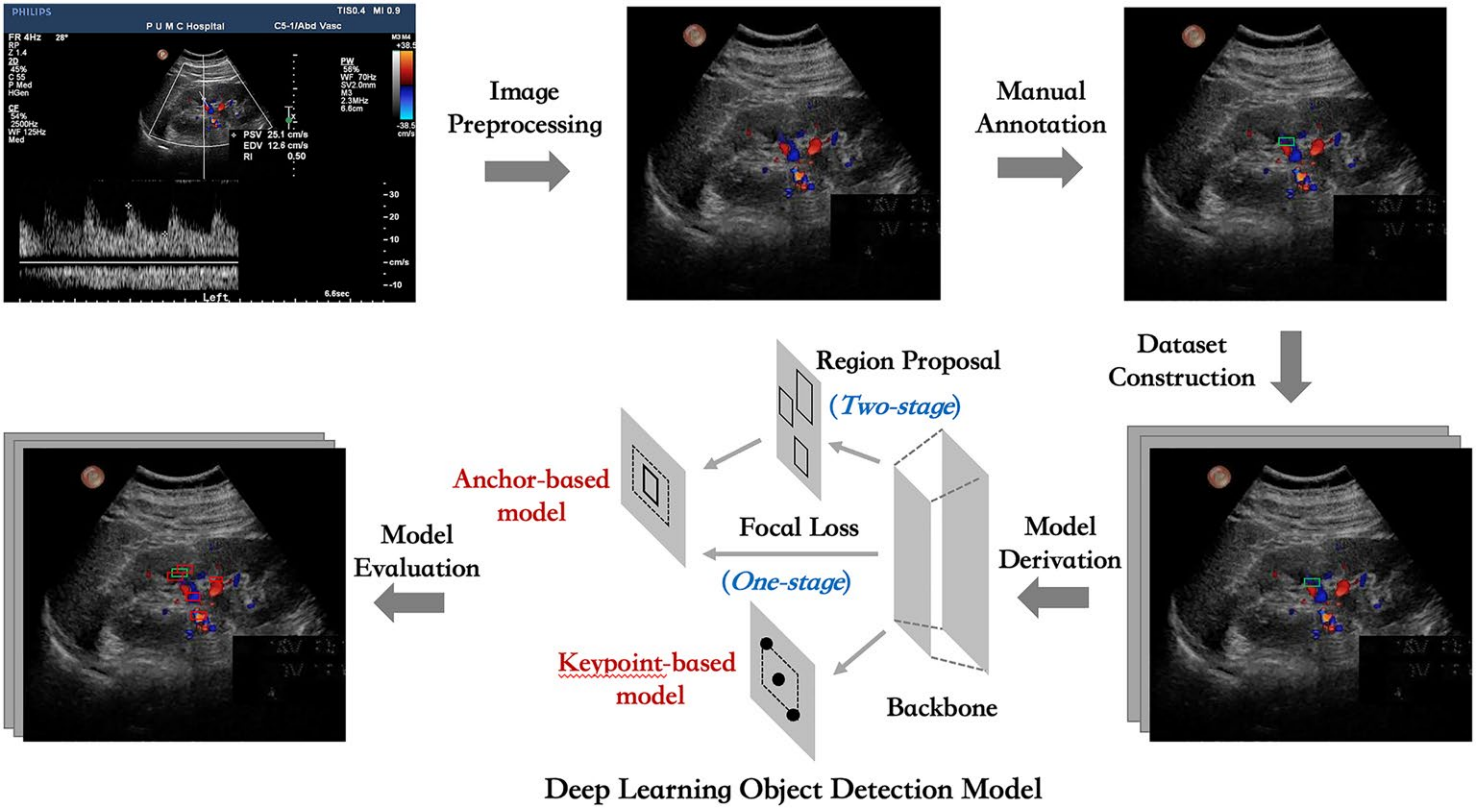
Schema of developing DL object detection models for CDS sampling position selection.

In this study, we proposed, to the best of our knowledge, for the first time, DL-based object detection approach to assist sampling position selection in the CDS images during renal artery ultrasound scanning (Fig. 3), by comparing predictive validities of multiple types of DL object detection models. A big retrospective ultrasound dataset was constructed and object detection models were evaluated comprehensively to conceptually verify the value of AI technology for renal artery ultrasound scanning.

## Materials and methods

### Subject and data

Patients with RAS who underwent renal artery ultrasound examinations performed by physicians with at least 3 years ultrasound experience at Peking Union Medical College Hospital between August 2017 and December 2019 were consecutively included. RAS (reduction of the lumen between 50 and 99%) was diagnosed based on specific criteria, including a peak systolic velocity (PSV) ≥ 150 cm/s in the renal artery, a ratio between PSV in the stenotic renal artery and PSV in the aorta (RAR) ≥ 2.5, or the presence of a tardus-parvus waveform^35–37^. Due to the low incidence rate of RAS and ultrasound being a screening method, the number of patients with RAS was considerably fewer than those without RAS. To address this, a comparable number of patients without RAS were randomly selected during the same time period.

The sampling position for AO was located 1 cm below the superior mesenteric artery. The scanning of the renal artery trunk included transverse scans of the upper abdomen, lateral coronal scans, and intercostal or subcostal transverse scans, with samples taken from the proximal, middle, and distal sections. The IRA were sampled in the upper, middle, and lower sections of the kidney. For vessels without stenosis, samples were taken from the center of the lumen, where blood flow is laminar. In the cases of narrowed vessels, samples were taken at locations with the most turbulent flow and highest PSV. All images were required to show clear anatomical structures, good color filling and proper sampling angles. Two sonographers reviewed the images, and only those meeting the specific criteria were included.

The study included a total of 873 patients with RAS and 1131 patients without RAS. The CDS images were categorized into four groups: AO, non-stenotic renal arteries (NRA), RAS, and IRA, based on the types of blood vessels. Basic statistical information for each category is presented in Table 1. The number of CDS images from both patient cohorts was comparable, with 4593 images from patients with RAS and 3722 images from patients without RAS. NRA and IRA images accounted for the majority, with 2878 and 3316 images, respectively, while the number of AO images was the lowest at 766. Additionally, the median area of the labeled sampling boxes for each type of CDS image was calculated (Table 1), with the RAS images having the smallest box area [70 (50, 99)].

**Table 1:** Characteristics of CDS images.

CDS image category	AO	NRA	RAS	IRA
Images from patients with RAS (N = 4593)	453	1047	1355	1738
Images from patients without RAS (N = 3722)	313	1831	0	1578
Sum (N = 8315)	766	2878	1355	3316
Sampling box area (pixels)	91 (55, 466.5)	170 (73.25, 315)	70 (50, 99)	96 (64, 156)
Height/width of sampling box	0.692 (0.56, 0.808)	0.75 (0.583, 1.0)	0.636 (0.5, 0.818)	0.846 (0.667, 1.133)

All CDS images were classified into four categories according to scanning position mainly. *CDS* color Doppler sonography, *AO* abdominal aorta, *NRA* normal renal artery, *RAS* renal artery stenosis, *IRA* intrarenal interlobular artery.

### Ethics declarations

This study was approved by the Institutional Review Board of the Peking Union Medical College Hospital (Ethical review number: JS-3306). All methods were conducted in accordance with the Declaration of Helsinki, and all participants provided informed consent.

### Image processing

The schema of data processing and model construction was shown in Fig. 3. DICOM files including both CDS image region and spectral waveform image region were selected and then pixel areas of CDS images were extracted. Then computer image processing algorithms were adopted to filter low-quality images with blur and color clutter, and to remove the device information, body mark, sampling frame and individual information in the original image. Next two physicians classified all images into four types, abdominal aorta (AO), normal renal artery (NRA), renal artery stenosis (RAS) and intrarenal interlobular artery (IRA) based on the types of blood vessels. After removing original sampling position information, images were annotated again and the sampling position of the original image were marked with tight rectangular box. The rectangular boxes were marked with the tool Make Sense (https://github.com/SkalskiP/make-sense). It should be noted that the sampling position prediction problem was simplified and the rotation angle of the sampling position was ignored in this study.

### Object detection model

**Figure 4:**
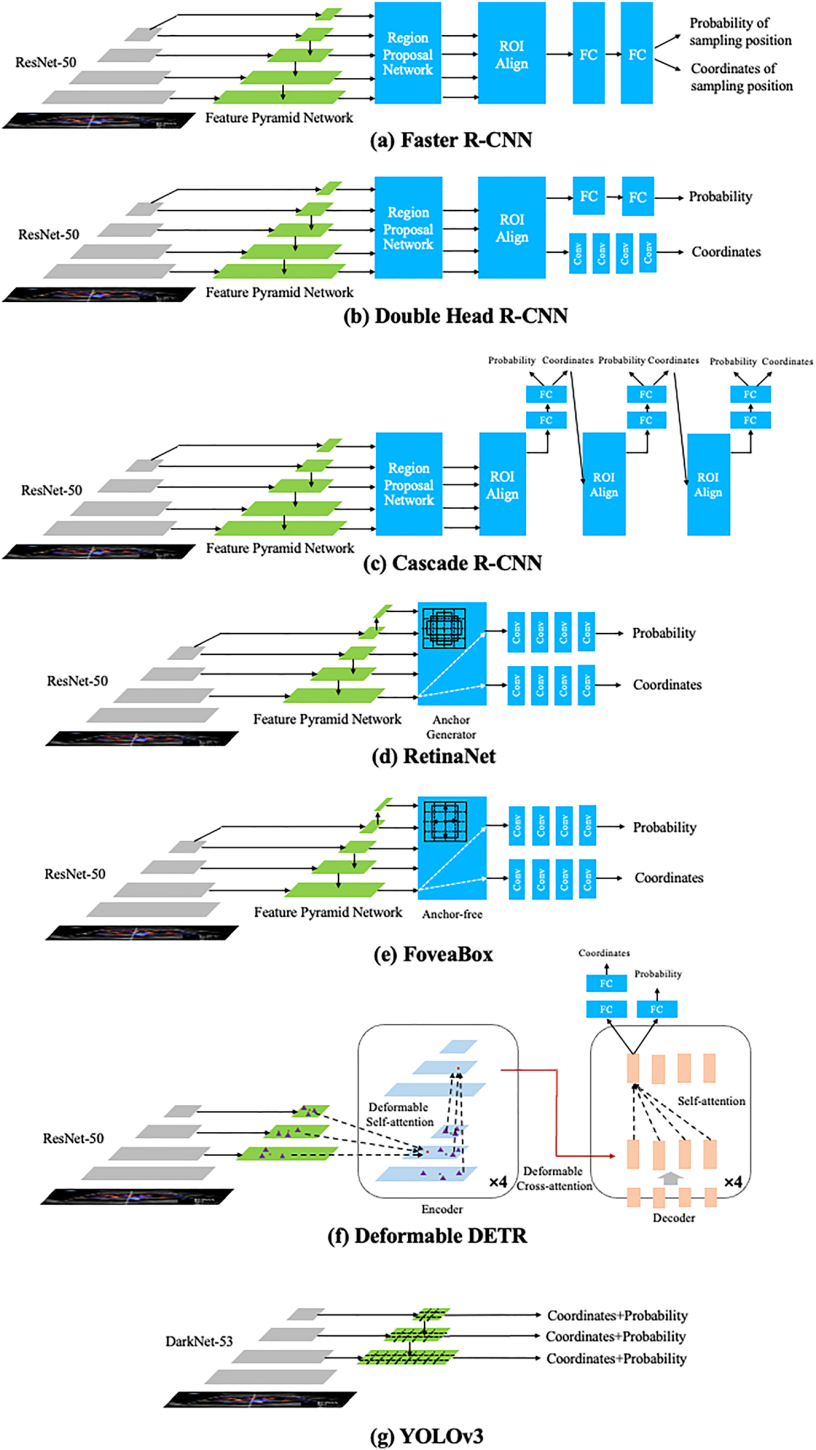
Illustration of architectures of seven DL object detection models.

The process of sampling position selection can be simplified and modeled using DL object detection technology to predict sampling rectangular box by analyzing patterns of anatomic structures and blood flow information in CDS images. Seven different DL object detection models were tested, including three two-stage models (Faster R-CNN^19^, Cascade R-CNN^20^, and Double Head R-CNN^21^) and four one-stage models (YOLOv3, RetinaNet, FoveaBox, and a Transformer-based model, Deformable DETR)^22–25^. The detailed architectures of seven models were shown in Fig. 4. Faster R-CNN model is a widely used object detection model and the proposed region proposal network (RPN) improves predictive performance greatly. The Cascade R-CNN includes multi-stage detector architecture to progressively enhance the quality of object detection. Double Head R-CNN adopts the fully connected network to predict category of object and the CNN structure to predict object’s location respectively, which enhances predictive precision. RetinaNet, utilizing multi-layer feature pyramid network (FPN) backbone and focal loss function to reduce the effect of class imbalance. YOLOv3 also adopted the idea of FPN and improved previous YOLO model. As a new anchor-free single-stage model, FoveaBox learns the object’s possibility and coordinates of boxes directly and shows comparable performance with the RetinaNet^24^. The Deformable DETR model combines the Transformer encoder-decoder architecture and the deformable attention to improve the training efficiency and achieved better performance than the DETR^38^.

After preprocessing, the input CDS image size was 450 × 350 pixels. The backbone of YOLOv3 was Darknet53, while other models utilized the ResNet-50 backbone and feature pyramid network (FPN) to extract five-level image feature information. The FPN outputted feature maps with 256 channels, and for single-stage models (RetinaNet and FoveaBox), the first level feature map from the ResNet-50 wasn’t used. For anchor-based models (Faster R-CNN, Double Head R-CNN, Cascade R-CNN, and RetinaNet), seven anchor ratios (0.33, 0.5, 0.75, 1.0, 1.33, 2.0, 2.5) were selected based on the statistical results of ratios of height and width of sampling positions (Table 1). In the region proposal networks (RPN) of two-stage object detection models (Faster R-CNN, Double Head R-CNN, and Cascade R-CNN), five anchor scales (8, 16, 20, 24, 32) and strides (4, 8, 16, 32, 64) were used. Three layers detection heads were used in Cascade R-CNN. For RetinaNet and FoveaBox, four convolution layers (Conv) were connected to predict both the probability and coordinates of sampling position. Similarly, Double Head R-CNN adopted 4 Convs to predict the coordinates. In the Deformable DETR, the dimension of embedding vectors was 256, the number of encoder and decoder layers was 6, and 300 object queries were used to predict sampling boxes.

### Model training and evaluation

During the training process, the positive samples’ fraction of RPN of three two-stage models was set to 25%, and learning rate was 0.0025. For each CDS image, the number of proposed results of RPN is limited to 1000. The learning rates of YOLOv3, FoveaBox, RetinaNet and Deformable DETR, were set to 1e−3, 1e−6, 1e−5, and 2 × 1e−4 respectively. The maximum number of iterations for all models was 30 epochs. To create the training dataset, 80% images of the CDS images of each type were randomly selected. Another 5% images were used to optimize the models’ parameters and select the best model, while the remaining 15% of the images comprised clinical validation dataset to evaluate the performance of assisting sampling position selection using the optimal model. It should be noted that all DL object detection models were trained to predict the sampling position and didn’t recognize the different image types.

**Figure 5:**
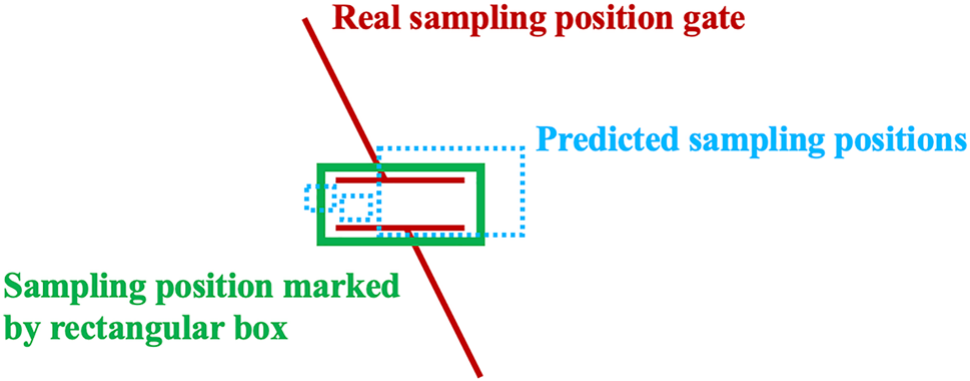
Comparison of predicted sampling positions and marked sampling position. Rectangular boxes with dotted blue lines were predicted results by model. The green rectangular box was labeled manually. The real sampling position gate was shown in red lines.

Due to the fact that the sampling position was usually small and was not unique in one CDS image, ultrasound physicians would select the most appropriate position from the candidate region according to their clinical experience. For example, for the sampling position of the normal intrarenal interlobar artery, multiple positions of the upper pole, the middle part and the lower pole could be selected. In addition, for the sampling position of large blood vessels such as the abdominal aorta, the sampling principle was to select the brightest place in the center of the blood vessel, which often corresponded to a small area within that region. The commonly used threshold criterion of intersection over union (IoU) ≥ 0.5 to judge the correctness of the predicted sampling box might be too strict. As shown in Fig. 5, for some predicted small sampling positions located inside the real sampling position or had big overlapping area with the manually marked rectangular box, they were also valuable indicators for the sonographer to select final sampling position.

In our experiments, instead of the IoU,

1 $$IoU=\frac{{Box}_{prediction}\cap {Box}_{target}}{{Box}_{prediction}\cup {Box}_{target}},$$

the self-overlap-ratio (SOR) was proposed by calculating the ratio of intersection area between the candidate sampling box and manually marked result as following,

2 $$SOR=\frac{{Box}_{prediction}\cap {Box}_{target}}{{Box}_{prediction}}.$$

When the value of SOR ≥ 0.5, the predicted sampling box was considered a match. If there was at least one matched box among top K predicted boxes with SOR ≥ 0.5, the predicted result by the object detection model was considered correct. The model’s accuracy was calculated as below,

3 $$Accuracy=\frac{\sum_{i=1}^{N}I\left[{\sum }_{k=1}^{K}I(SOR\ge 0.5) >0\right]}{N}$$

where $I\left(x>0\right)=1$ is the indicator function, the N was the number of samples, and the K = 10 was the number of top predicted sampling positions in each image sorted by their object probabilities (> 0.5) in decreasing order.

### Model implementation and statistical analysis

In this study, image processing was done with Python3.7.6 and computer vision library opencv. DL object detection models were constructed with PyTorch 1.4.0, torchvision package, and the deep learning object detection library, MMDetection^39^. The normally distributed variables were denoted as mean values with standard deviations, and categorical variables were formatted with counts and proportions. Ten trained results of every DL model were saved, and difference of predictive accuracies of different models were compared by the hypothesis testing. Independent two-sample *t*-test was used for variables obeying the normal distribution. The threshold for statistical significance was set to 0.05. All statistical analysis was conducted using IBM SPSS software (version 26.0, International Business Machines Corp., Chicago, IL, USA) (https://www.ibm.com/support/pages/downloading-ibm-spss-statistics-26).

## Results

The predictive performances of seven models on the parameter optimization dataset and clinical validation dataset were presented in Table 2. Compared to the results obtained on the parameter optimization dataset, the accuracies of the seven models on the validation dataset showed a relative reduction. Among the seven models, Double Head R-CNN exhibited the highest average accuracies on both datasets (89.3% on the parameter optimization dataset and 88.5% on the clinical validation dataset), clearly outperforming the other models (P < 0.001). Besides, on the validation dataset, Double Head R-CNN achieved the best accuracy for each type of CDS image (P < 0.001). The predictive accuracies of Faster R-CNN (78.5% and 77.3%) and the mean accuracies of Cascade R-CNN (79.5% and 77.1%) were similar (P = 0.684). The performances of the single-stage object detection model, RetinaNet (58.5% and 56.5%), the anchor-free model, FoveaBox (52.9% and 50.7%), and the NLP-based model, Deformable DETR (66.6% and 65.4%), were significantly lower than the two-stage models (P < 0.001). The predictive validity of FoveaBox was inferior to that of RetinaNet (P = 0.004). On the clinical validation dataset, the accuracies of Deformable DETR surpassed those of FoveaBox and RetinaNet (P < 0.001). However, the predictive accuracy of YOLOv3 (85.5% and 84.2%) was higher than most detectors, except for Double Head R-CNN, including the remaining single-stage detectors and the two two-stage models (Faster R-CNN and Cascade R-CNN; P < 0.001).

**Table 2:** Comparison of predictive accuracies of seven DL object detection models.

Object detection method	Parameter optimization dataset	Clinical validation dataset	AO	NRA	RAS	IRA
Double Head R-CNN	89.3 ± 0.6%	88.5 ± 0.3%	86.5 ± 1.1%	90.4 ± 0.1%	84.7 ± 1.0%	88.8 ± 0.6%
Faster R-CNN	78.5 ± 1.6%	77.3 ± 1.2% (P < 0.001)	69.8 ± 2.2%	82.6 ± 1.1%	68.1 ± 2.5%	78.2 ± 0.9%
Cascade R-CNN	79.5 ± 1.2%	77.1 ± 1.2% (P < 0.001)	70.4 ± 2.2%	80.6 ± 1.0%	68.2 ± 2.3%	79.2 ± 1.5%
RetinaNet	58.5 ± 3.0%	56.5 ± 2.6% (P < 0.001)	45.5 ± 1.8%	66.5 ± 1.9%	36.7 ± 3.3%	58.4 ± 3.4%
YOLOv3	85.5 ± 2.7%	84.2 ± 1.6% (P < 0.001)	80.6 ± 2.4%	86.9 ± 1.6%	75.6 ± 2.8%	86.2 ± 1.8%
FoveaBox	52.9 ± 1.3%	50.7 ± 1.5% (P < 0.001)	38.4 ± 0.8%	62.1 ± 1.5%	28.7 ± 2.8%	52.7 ± 1.8%
Deformable DETR	66.6 ± 4.5%	65.4 ± 3.8% (P < 0.001)	55.8 ± 4.3%	71.1% ± 3.2%	45.7 ± 5.9%	70.7 ± 4.1%

Moreover, the comprehensive accuracies of the optimal model, Double Head R-CNN, on the four categories of CDS images are presented in Table 3. The results demonstrate that the prediction accuracies for NRA (92.1%) and IRA (90.9%) were higher compared to those for AO (86.8%) and RAS (85.1%) on the clinical validation dataset, which aligns with the pattern observed in the validation dataset from Table 2 (P < 0.001). Generally, the accuracies for the four categories of images on the validation dataset decreased compared to the training dataset or the parameter optimization dataset. The predictive accuracies for NRA and IRA reduced by approximately 4%, while the results for AO and RAS decreased by approximately 5%.

**Table 3:** Predictive accuracies of Double Head R-CNN model on three datasets.

Image category	Training dataset	Parameter optimization dataset	Clinical validation dataset
AO	92.2% (567/615)	86.5% (32/37)	86.8% (99/114)
NRA	95.7% (2205/2304)	94.4% (135/143)	92.1% (397/431)
RAS	89.6% (973/1086)	83.6% (56/67)	85.1% (172/202)
IRA	95.3% (2531/2656)	93.3% (153/164)	90.9% (451/496)

**Figure 6:**
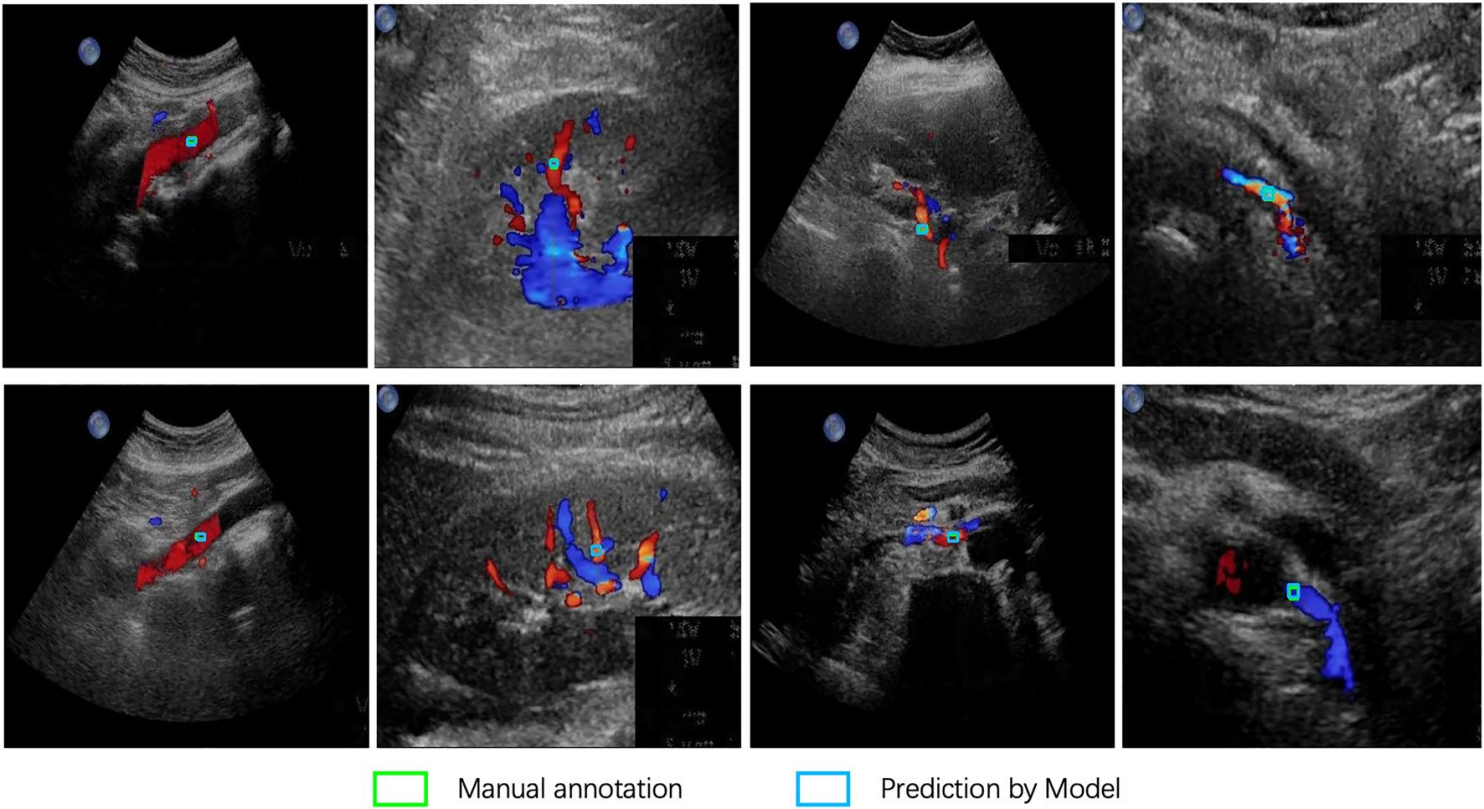
Examples of predicted sampling positions by the Double Head R-CNN model for different types of CDS images.

In Fig. 6, examples of predicted sampling positions by the optimal object detection model on the clinical validation dataset are illustrated. Only the sampling position with the highest probability is plotted with a blue rectangular box. It is evident that the predicted results closely align with the manually labeled sampling positions, illustrating that the Double Head R-CNN model effectively learned the pattern of sampling position selection.

## Discussion

During renal artery ultrasound scanning, the selection of the sampling position on CDS images is an important step. In this study, we tried to simplify this process by treating it as an object detection task, utilizing a DL model to analyze image information and locate suitable sampling boxes. By comparing predictive performance of seven DL objection detection models on CDS images datasets, we found that the anchor-based two-stage object detection model, Double Head R-CNN, achieved a promising accuracy of 88.5 ± 0.3% on the clinical validation dataset, indicating the potential of DL object detection models in assisting sampling position selection. The models developed in this study are valuable in assisting inexperienced operators in choosing appropriate sampling positions and avoiding misdiagnosis. This study serves as a necessary and fundamental step towards the realization of AI-assisted renal artery ultrasound scanning and the diagnosis of RAS.

Significant differences in predictive efficiency were observed among the various object detection models tested in this study. As shown in Table 2**,** all two-stage object detection models and three one-stage models (RetinaNet, YOLOv3, and Deformable DETR) outperformed the anchor-free model, FoveaBox (50.7 ± 1.5%; P < 0.001). Additionally, the two-stage anchor-based models (Double Head R-CNN, Faster R-CNN, and Cascade R-CNN) demonstrated superior performance compared to RetinaNet (56.5 ± 2.6%) and Deformable DETR (65.4 ± 3.8%; P < 0.001). While keypoint-based DL object detection models have gained popularity due to their simplicity, they may not be suitable for assisting sampling position selection, as evidenced by the significantly lower accuracies of FoveaBox compared to other models. Notably, FoveaBox achieved an accuracy of only 28.7 ± 2.8% on RAS images, which was substantially lower than the other models (P < 0.001). Furthermore, although RetinaNet's performance (56.5%) was higher than that of the keypoint-based model (50.7%) on the validation dataset (P < 0.001), its predictive validity still fell short compared to Cascade R-CNN (77.1 ± 1.2%; P < 0.001). Among the four one-stage models, YOLOv3 achieved the best predictive results and ranked second after Double Head R-CNN. In summary, anchor-based two-stage models and YOLOv3 were deemed more suitable for sampling position prediction.

The three single-stage models (RetinaNet, FoveaBox, and Deformable DETR) differ from the two-stage models in their approach to handling low-confidence sampling positions. Unlike the two-stage models, they do not employ the RPN network (Fig. 4) to filter out such positions initially, but instead process all possible sampling positions, including numerous negative cases. This increases the difficulty of object detection. Additionally, in order to reduce the training time and memory requirements, these models don’t utilize the image feature information from the first block of ResNet-50 backbone ($\frac{1}{{4}^{2}}$ area of original CDS image). Consequently, they struggle to capture fine-grained image feature, resulting in low prediction accuracy. In contrast, the predictive accuracies of Cascade R-CNN and Faster R-CNN were similar (P = 0.684). Compared with Faster R-CNN, the only difference of Cascade R-CNN is the multi-stage detectors based on the proposed sampling positions by the RPN module. However, the sampling position selection relies more on the analysis of surrounding anatomic structures and blood flow patterns, this characteristic of Cascade R-CNN doesn’t contribute significantly to improving the models’ performance. Unlike other models, Double Head RCNN model utilizes multiple convolutional layers to predict the coordinates of sampling positions, instead of the full connected layers, making it a better choice for sampling position prediction. Further we compared the performance of Double Head R-CNN with three recently published models (DINO^40^, DAB-DETR^41^, and RTMDet^42^), and predictive accuracies of them were listed at Supplementary Table 1. These three models’ accuracies were still lower than the Double Head R-CNN.

Although DL models have demonstrated excellent performance in classical object detection problems such as human face detection and have been widely deployed in various applications, their effectiveness in sampling position prediction (Tables 2 and 3) is not as good as that in face detection. This discrepancy can be attributed to the differences between traditional object detection and ultrasound sampling position detection (as depicted in Fig. 1). The similarity between a sampling position and its surrounding region, as well as the reliance on overall analysis of anatomical structures and blood flow information in the CDS image, pose challenges for current DL technologies to model accurately, leading to a decrease in predictive validity. Convolutional neural networks typically assume a local receptive field to reduce the complexity of the model architecture. Consequently, capturing global information from the image necessitates a larger number of convolutional layers. However, increasing the number of max pooling operations or using large strides in convolutional neural networks reduces the resolution of feature maps, which in turn blurs subtle image features and hinders the accurate localization of sampling positions. All object detection models used ResNet-50 or DarkNet-53 backbone and FPN to extract image feature information, and sizes of four feature maps outputted by the ResNet-50 are the $\frac{1}{{4}^{2}}$, $\frac{1}{{8}^{2}}$, $\frac{1}{{16}^{2}}$, and $\frac{1}{{32}^{2}}$ of original CDS image, making it difficult for DL model to locate the small sampling position precisely. Other DL models which are not based on the convolutional neural network should be designed and improved further.

Compared with previous ultrasound image studies based on object detection technology (Fig. 1), this study provides a comprehensive evaluation of various DL object detection models for sampling position selection to find the optimal model. The FPN module is incorporated into seven models to capture the multi-scale image feature information. Except for YOLOv3, the performance of most of single stage models (RetinaNet, FoveaBox and Deformable DETR) didn’t exceed the two-stage models (77.1 ± 1.2%) on CDS sampling position selection problem. The experiment results are similar to those of Lin et al. and Bassiouny et al.^25,30^ indicating that anchor-based two-stage models outperform most of the single stage models (P < 0.001). Different from previous studies that only tested Faster R-CNN model, this study further compares three two-stage models (Double Head R-CNN, Faster R-CNN and Cascade R-CNN). The Double Head R-CNN achieved the optimal predictive accuracy (88.5 ± 0.3%; P < 0.001). However, our experiment results weren’t consistent with the Cao et al. and Dadoun et al.^29,34^. In the study on breast lesion^29^, only 579 benign and 464 malignant lesion ultrasound images were used for training the models, and may lead to insufficient assessment of the significance of differences among DL models. For the study on focal liver lesion^34^, it may be the reason that the texture of the lesions differs more from the surrounding tissue compared to the sampling position in CDS ultrasound images. This distinction may make it easier for the Transformer-based model, DETR, to recognize the lesions.

The above-mentioned DL models’ accuracies were calculated using the SOR criteria instead of the IoU. We further compared the differences between these two criteria on sampling position prediction. From Table 4, models’ accuracies with the SOR on three datasets (training, parameter optimization, and clinical validation datasets) were relatively higher than the results obtained using the IoU. This is mainly due to the SOR criterion being more lenient in determining positive samples. As the number of sampling positions with high probabilities (K) increased, the models' performance gradually improved. In our experiments, K was set to 10 to compare the efficiency of different DL models, but other values of K can be selected during model deployment. When the value of K was changed from 10 to 4, the accuracy of the model's predictions decreased by approximately 4% for parameter optimization and clinical validation data. The predictive performance of the Double Head R-CNN model with K = 4 on the four types of images is further presented in Table 5.

**Table 4:** Difference of models’ accuracies by using the SOR or IoU criteria.

Metrics	Top@K = 1	K = 2	K = 4	K = 6	K = 8	K = 10	K = 14	K = 18	K = 20
SOR (train)	0.833	0.911	0.931	0.937	0.940	**0.942**	0.944	0.946	0.946
SOR (optimization)	0.679	0.810	0.873	0.893	0.903	**0.915**	0.927	0.929	0.929
SOR (clinical)	0.664	0.808	0.864	0.880	0.891	**0.900**	0.908	0.912	0.913
IoU (train)	0.723	0.861	0.896	0.908	0.915	**0.919**	0.923	0.926	0.926
IoU (optimization)	0.433	0.620	0.727	0.769	0.800	**0.825**	0.859	0.869	0.869
IoU (clinical)	0.465	0.654	0.745	0.784	0.805	**0.825**	0.845	0.852	0.854

The K is the selected number of top predicted sampling positions. SOR (train) and IoU (train) means the SOR or IoU values on the training dataset. The K = 10 was used to compare DL models’ performance. The results were based on the Double Head R-CNN model. Significant values are in bold.

**Table 5:** Predictive accuracies of Double Head R-CNN model with K = 4.

Image category	Training dataset	Parameter optimization dataset	Clinical validation dataset
AO	91.5% (563/615)	78.4% (29/37)	85.1% (97/114)
NRA	94.4% (2174/2304)	92.3% (132/143)	87.5% (377/431)
RAS	87.6% (951/1086)	77.6% (52/67)	82.7% (167/202)
IRA	94.7% (2515/2656)	89.0% (146/164)	87.3% (433/496)

Some limitations are present in this study that should be acknowledged. Firstly, the number of RAS images and AO images is relatively small. When we trained the object detection model with a class-balanced dataset by using equal number (N = 615) of four types of CDS images, the model’s accuracy decreased dramatically (Supplementary Table 2). To address the class imbalance of the four types of CDS images, it is necessary to include more patients and construct multi-center cohorts to better validate the effectiveness of DL models. Additionally, the process of eliminating the original sampling gates using a computer algorithm may introduce biases. CDS images without sampling gates are more suitable for model construction by collecting ultrasound scanning videos. Thirdly, our study simplified the process of sampling position selection, and the rotation of the sampling position box should be considered in further research. Furthermore, in renal artery ultrasound, examinations, image clarity is crucial as it serves as the foundation for all subsequent measurements and analyses. In the future, we aim to achieve AI-assisted scanning and diagnosis, including obtaining clearer images, implementing automatic measurement and diagnosis algorithms.

## Conclusion

This study serves as a proof of concept for the application of DL object detection models in assisting CDS sampling position selection during renal artery ultrasound examinations. Different types of object detection models were compared in detail and the anchor-based two-stage model, Double Head R-CNN, achieved an average accuracy of 88.5% on clinical validation images and outperformed the others. These findings indicate the potential integration of DL object detection models into routine ultrasound scanning procedures, providing assistance to inexperienced physicians and improving the accuracy of sampling position selection.

### Supplementary Information

Supplementary Tables.

## Data Availability

Data are available upon reasonable request to the corresponding author.
