# Supplementary material for: Deep-learning-based sampling position selection on color Doppler sonography images during renal artery ultrasound scanning
Source: Sci Rep. 2024 May 23;14:11768. doi: 10.1038/s41598-024-60355-5 (PMC11116437; doi:10.1038/s41598-024-60355-5)
Supplement: Supplementary file 1 — Supplementary Tables. [file 41598_2024_60355_MOESM1_ESM.docx]

**Supplementary Table 1. Comparisons of predictive accuracies between the Double Head R-CNN and three recently published methods.**

| **Object detection method** | **Parameter optimizing dataset** | **Clinical validation dataset** | **AO** | **NRA** | **RAS** | **IRA** |
| --- | --- | --- | --- | --- | --- | --- |
| Double Head R-CNN | 89.3±0.6% | 88.5±0.3% | 86.5±1.1% | 90.4±0.1% | 84.7±1.0% | 88.8±0.6% |
| DINO | 86.7±4.3% | 84.9±3.6% | 81.9±5.7% | 88.3±3.1% | 78.4±6.1% | 85.4±2.9% |
| DAB-DETR | 51.6±5.2% | 50.8±4.8% | 46.3±3.2% | 62.1±4.7% | 32.3±7.0% | 49.6±3.2% |
| RTMDet | 58.7±7.2% | 58.8±8.7% | 51.1±10.6% | 65.7±5.6% | 38.4±14.1% | 62.8±8.9% |

Three models included one one-stage model, RTMDet, and two end-to-end models with encoder-decoder architectures, DINO and DAB-DETR.

**References**

1. Lyu, Chengqi, et al. " RTMDet: An empirical study of designing real-time object detectors." arXiv preprint arXiv:2212.07784 (2022).
2. Zhang, Hao, et al. "DINO: Detr with improved denoising anchor boxes for end-to-end object detection." arXiv preprint arXiv:2203.03605 (2022).
3. Liu, Shilong, et al. " DAB-DETR: Dynamic anchor boxes are better queries for detr." arXiv preprint arXiv:2201.12329 (2022).

**Supplementary Table 2. Comparison of predictive accuracies of Double Head R-CNN trained with full dataset or class-balanced dataset.**

| **Dataset Type** | **Parameter optimizing dataset** | **Clinical validation dataset** | **AO** | **NRA** | **RAS** | **IRA** |
| --- | --- | --- | --- | --- | --- | --- |
| Full | 89.3±0.6% | 88.5±0.3% | 86.5±1.1% | 90.4±0.1% | 84.7±1.0% | 88.8±0.6% |
| Balanced | 76.1±1.5% | 76.6±1.7%(P<0.001) | 68.9±3.0% | 82.9±1.6% | 66.2±2.4% | 77.2±1.9% |

For the class-balanced dataset, the number of four types of CDS images is the same (N=615).
